# Segmented jaw‐locking in IMRT for upper thoracic esophageal cancer: A plan complexity‐driven approach to lung dose reduction

**DOI:** 10.1002/acm2.70467

**Published:** 2026-01-15

**Authors:** Shi Cao, Guang‐Zhi Sun, Jun‐Yi Gao, Xiang Dai, Hao Wang, Chao‐Min Chen, Wan‐Song Xu, Yi‐Hai Fang

**Affiliations:** ^1^ Department of Radiation Oncology Taizhou People's Hospital Affiliated to Nanjing Medical University Taizhou Jiangsu China; ^2^ Department of Radiology Taizhou People's Hospital Affiliated to Nanjing Medical University Taizhou Jiangsu China; ^3^ Institute of Medical Instruments School of Biomedical Engineering Southern Medical University Guangzhou Guangdong China; ^4^ Department of Medical Engineering Zhujiang Hospital Southern Medical University Guangzhou Guangdong China

**Keywords:** elective nodal irradiation (ENI), lung dose, plan complexity, segmented jaw‐locking IMRT (SJL‐IMRT), upper thoracic esophageal cancer

## Abstract

**Objective:**

Extended‐field intensity‐modulated radiotherapy (EF‐IMRT) used for elective nodal irradiation (ENI) in upper thoracic esophageal cancer frequently results in excessive intermediate‐to‐low‐dose pulmonary irradiation. This study presented a practical and reproducible planning strategy (segmented Jaw‐locking IMRT, SJL‐IMRT) for optimizing dose distributions to ENI target volumes, and assessed its impact on plan complexity and lung parenchyma sparing.

**Methods:**

In a paired planning study (*n* = 40), EF‐IMRT and SJL‐IMRT plans were generated per patient under identical target coverage objectives. SJL‐IMRT partitioned the longitudinal ENI volume into cervical‐supraclavicular and upper‐mediastinal segments via coordinated orthogonal collimators, and multi‐leaf collimator (MLC)‐defined apertures locked within each segment. The evaluation was based on multifaceted criteria, including metrics for: (i) plan complexity: the aperture‐based edge‐area metric (EAM) and the sequence‐level modulation complexity score (MCS); (ii) dosimetry: conformity index (CI), homogeneity index (HI), gradient measure (GM), pulmonary parameters (mean lung dose [MLD], V_5_∼V_30_), and spinal cord maximum dose (D_max_); (iii) radiobiological effects: tumor control probability (TCP) and normal tissue complication probability (NTCP). The delivery accuracy of SJL‐IMRT and EF‐IMRT was validated with a PTW OCTAVIUS 729 2D ionization chamber array and RW3 phantom, using *γ* analysis (3.0%/3.0 mm criterion, global normalization, 10% dose threshold). Statistical analysis was performed using Wilcoxon signed‐rank test.

**Results:**

Both SJL‐IMRT and EF‐IMRT satisfied prescription dose objectives for planning target volume (PTV). No significant differences were observed in CI and HI (*p *= 0.347 and *p *= 0.173, respectively). SJL‐IMRT demonstrated lower geometric complexity and simpler sequencing: EAM 8.610 ± 2.951 vs. 20.824 ± 4.944 (paired Δ = −12.214; *p *= 0.00195) and MCS 0.243 ± 0.015 vs. 0.203 ± 0.036 (paired Δ = +0.040; *p *= 0.0193), respectively. Compared with EF‐IMRT, SJL‐IMRT (i) reduced gradient measures (GM) by 0.215 cm (*p <* 0.01), indicating a steeper dose fall‐off; (ii) decreased MLD by 1.62 Gy (left) and 2.64 Gy (right); (iii) lowered left lung V_5_ and V_20_ by 19.96%, 3.63%, right lung V_5_ and V_20_ by 25.27%, 3.05%, respectively (all *p <* 0.01); (iv) exhibited a marginally higher mean *γ* passing rate (99.4 ± 0.72% vs. 98.8 ± 0.75%, *p *= 0.048), no dose cold/hot spots in the 2‐cm feathered overlap, and a more compact high‐dose area; (v) demonstrated a longer mean delivery time (10.9 vs. 8.8 min, *p *= 0.021) and higher total monitor units (MUs: 2893 vs. 2066; *p *= 0.026). (vi) translated these dosimetric gains into significant NTCP reductions for both lungs (left −1.30%, right −1.29%; *p <* 0.01). TCP values were numerically higher for SJL‐IMRT, but these differences were not statistically significant; D_max_ to the spinal cord followed the same pattern.

**Conclusion:**

SJL‐IMRT reduces plan complexity (EAM↓, MCS↑) and lowers intermediate‐to‐low lung dose compared with EF‐IMRT for ENI in upper thoracic esophageal cancer, without compromising target coverage, supporting SJL‐IMRT as a pragmatic approach to improving dosimetric quality and delivery simplicity. Confirmation in larger cohorts is warranted.

## 1 INTRODUCTION

Esophageal cancer represents a prevalent malignancy of the digestive tract, with tumors originating in the upper thoracic region (cervical/upper thoracic segments, ≤24 cm from incisors) comprising approximately 14%–15% of all cases.^1^, ^2^ Surgical management in the upper thoracic region is technically challenging because of its proximity to critical laryngopharyngeal structures. Moreover, the rich vascular and lymphatic networks further facilitate early hematogenous and lymphatic dissemination of tumors. Consequently, definitive radiotherapy has become the preferred treatment modality for upper thoracic esophageal cancer, providing satisfactory oncologic control while preserving organ function and quality of life.^3^, ^4^ The primary lymphatic drainage of the upper thoracic esophagus involves bilateral supraclavicular and upper‐middle mediastinal nodal basins, elective nodal irradiation (ENI) is therefore routinely employed to eradicate subclinical metastases and improve locoregional control.^5^, ^6^ However, the extensive and geometrically complex ENI target volumes inevitably increase radiation exposure to adjacent organs at risk (OARs), most notably the lungs, heart, and spinal cord.^7^ Radiation pneumonitis (RP), the principal dose limiting pulmonary toxicity, can progress to emphysema, fibrosis, and chronic inflammation, markedly degrading quality of life.^8^ A strong dose‐response relationship has been documented between RP incidence and low‐dose lung metrics, and keeping lung V_5 _< 55% and V_20 _< 25% markedly reduces ≥Grade 2 radiation pneumonitis, underscoring the clinical imperative to minimize low‐dose pulmonary irradiation in this patient population.^9^, ^10^ Similarly, spinal cord $D_{\max}$ must remain below 45 Gy to avoid late myelopathy.^11^

Conventional IMRT often struggles to reconcile high‐dose conformity with stringent OARs sparing when treating the elongated and irregular ENI volume of upper thoracic esophageal cancer. Target coverage must span the deep cervical chains, supraclavicular fossae, and upper mediastinum, forcing full jaw opening of the linear accelerator and extensive dynamic motion of the multi‐leaf collimator (MLC). Such longitudinally extensive modulation is associated with higher monitor units, greater opportunity for interleaf transmission and tongue‐and‐groove effects, resulting in an expanded intermediate‐to‐low‐dose “bath” to adjacent OARs, most notably the lungs.^12^ To address the above limitations, we implemented a segmented Jaw‐locking IMRT (SJL‐IMRT) strategy for ENI. In SJL‐IMRT, the collimator jaws divide the longitudinal irradiation volume into distinct cervical‐supraclavicular and upper‐mediastinal subfields, and the MLC‐defined apertures are locked within each segment.

This design constrains the active longitudinal span, eliminates non‐contributory open rows, and is intended to regularize the aperture geometry and smooth leaf trajectories. A feathered overlap (2 cm) across the segment junction produces a continuous dose gradient, avoiding junctional hot sports or cold spots. Conceptually, aperture locking acts as a geometric regularizer that should reduce plan complexity and, by limiting unnecessary edge formation, curtail low‐dose spread to lung parenchyma while preserving prescription coverage.

## 2 MATERIALS AND METHODS

### 2.1 Clinical data

This retrospective study analyzed data from 40 patients with histologically proven upper thoracic esophageal cancer treated with radiotherapy between March 2023 and January 2024 at Taizhou People's Hospital affiliated to Nanjing Medical University. All tumors originated in cervical or upper thoracic esophageal segments (≤24 cm from incisors), with no evidence of distant metastasis (M0). The cohort comprised 20 males and 20 females, with a median age of 69 years (range 41–85). Complete clinical and imaging data were available for all enrolled patients. Inclusion criteria were:

1. Primary tumor located ≤24 cm from incisors (cervical/upper thoracic esophagus).
2. Clinical stage T1‐4N0‐3M0 (AJCC 8th edition).
3. No absolute contraindications to radiotherapy.
4. ECOG performance status 0–2 with life expectancy≥3 months.

Exclusion criteria were:

1. Prior thoracic radiotherapy or esophageal resection.
2. Severe cardiopulmonary dysfunction (FEV_1_ < 50% predicted or LVEF < 40%).
3. Active connective tissue disease or interstitial lung disease.

### 2.2 CT simulation

All patients underwent free‐breathing CT simulation in the supine position using a flat‐top couch. The upper extremities were positioned alongside the torso, with individualized thermoplastic masks immobilizing the head, neck, and upper thorax to minimize positioning uncertainties. Following intravenous administration of 60 mL iodinated contrast medium, helical CT scans were acquired from the C3 vertebral level to 2 cm below the diaphragmatic dome, fully encompassing the lungs, at 3 mm slice thickness. All DICOM datasets were exported to the Varian Eclipse treatment planning system for target and organs‐at‐risk (OARs) delineation and heterogeneity‐corrected dose calculation.

### 2.3 Target volume and OARs delineation

Target volumes of the upper thoracic esophageal cancer were delineated according to ICRU Report 83 definitions: gross tumor volume (GTV), clinical target volume (CTV), and planning target volume (PTV).^13^, ^14^ Contouring integrated multiparametric evidence (contrast‐enhanced CT, endoscopic findings), lymphatic drainage patterns, and spatial relationships to OARs to ensure target coverage while minimizing irradiation of normal tissues.

#### 2.3.1 GTV

The GTV comprised two components:

**GTV‐T**: Primary tumor identified on free‐breathing CT as asymmetric wall thickening (>5 mm), luminal distortion, or abnormal soft‐tissue density.
**GTV‐N**: Metastatic lymph nodes defined by short‐axis diameter ≥10 mm (mediastinal) or ≥5 mm (supraclavicular), irregular enhancement. Special attention was given to radiographically suspicious cervical, supraclavicular, and upper mediastinal nodal regions.

#### 2.3.2 CTV

CTV boundaries respected natural anatomical barriers: trachea, vertebral bodies, and pleura.

**CTV‐T**: GTV‐T+3 cm longitudinal margin (accounting for submucosal spread) +1 cm radial margin.
**CTV‐N**: GTV‐N+0.5 cm uniform margin and bilateral supraclavicular (level IV), upper paratracheal (stations 2R/2L/4R/4L), subcarinal nodal.

#### 2.3.3 PTV

**FIGURE 1:**
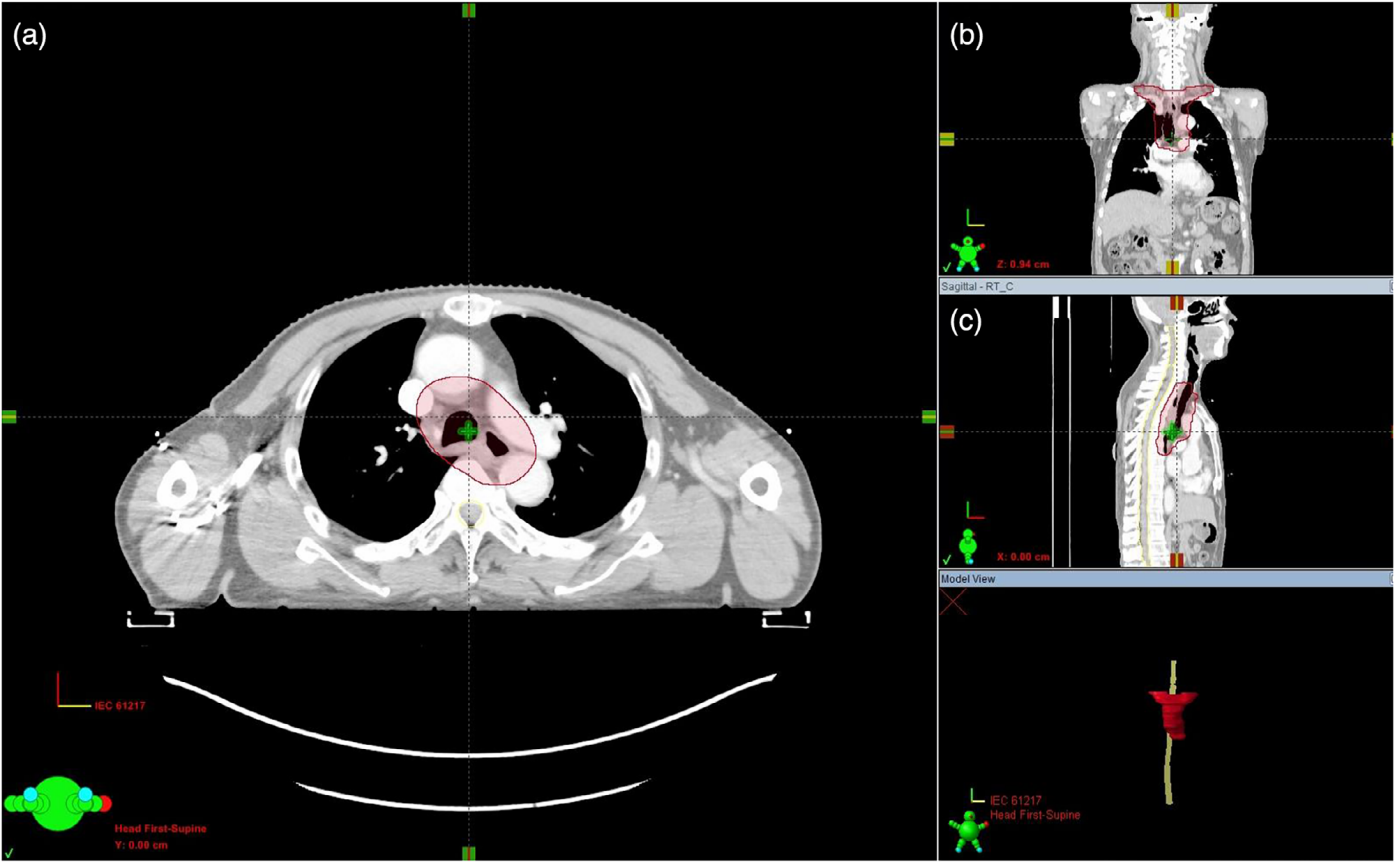
Radiotherapy PTV for upper esophageal cancer. A: Transverse section, B: coronal section, C: sagittal section.

The PTV was generated through anisotropic expansion of the CTV to account for patient positioning errors, respiratory motion, and mechanical uncertainties during treatment delivery (Figure 1).

### 2.4 Treatment planning and delivery

Radiotherapy plans were generated in Eclipse TPS and delivered with a Varian Trilogy linac using 6‐MV photon beams. The machine's Millennium 120 MLC comprises 60 leaf pairs (40 central pairs projecting 5 mm and 20 peripheral pairs projecting 10 mm at isocenter), allowing a maximum field size of 40 × 40 cm^2^. Dynamic jaw tracking is not available on this platform, and jaw positions are fixed during each beam at the values determined during planning. Two static‐gantry IMRT techniques were developed for each patient: EF‐IMRT and SJL‐IMRT. A total dose of 50 Gy in 25 fractions (2 Gy per fraction) was prescribed to ensure that ≥ 95% of the planning target volume (PTV) received ≥ 95% of the prescription dose. Optimization priorities were: (i) Primary goal: ≥ 98% of the clinical target volume (CTV) covered by ≥ 95% of the prescription. (ii) Secondary goal: adherence to oragan‐at‐risk constraints (Table 1).

**TABLE 1:** Adherence to organ‐at‐risk constraints in radiotherapy for upper thoracic esophageal cancer.^15^

OARs	Dose constraint
Lung	V_5_ ≤ 55% V_20_ ≤ 25% V_30_ ≤ 20% MLD ≤ 13 Gy
Spinal cord	D_max_≤ 45 Gy
Heart	MHD ≤ 26 Gy V_30_ ≤ 40% V_40_ ≤ 30%

*Note*: V_x_, percentage volume of organ receiving at least x Gy; MLD, mean lung dose; D_max_, maximum dose; MHD, mean heart dose.

#### 2.4.1 Extended‐field IMRT (EF‐IMRT)

**FIGURE 2:**
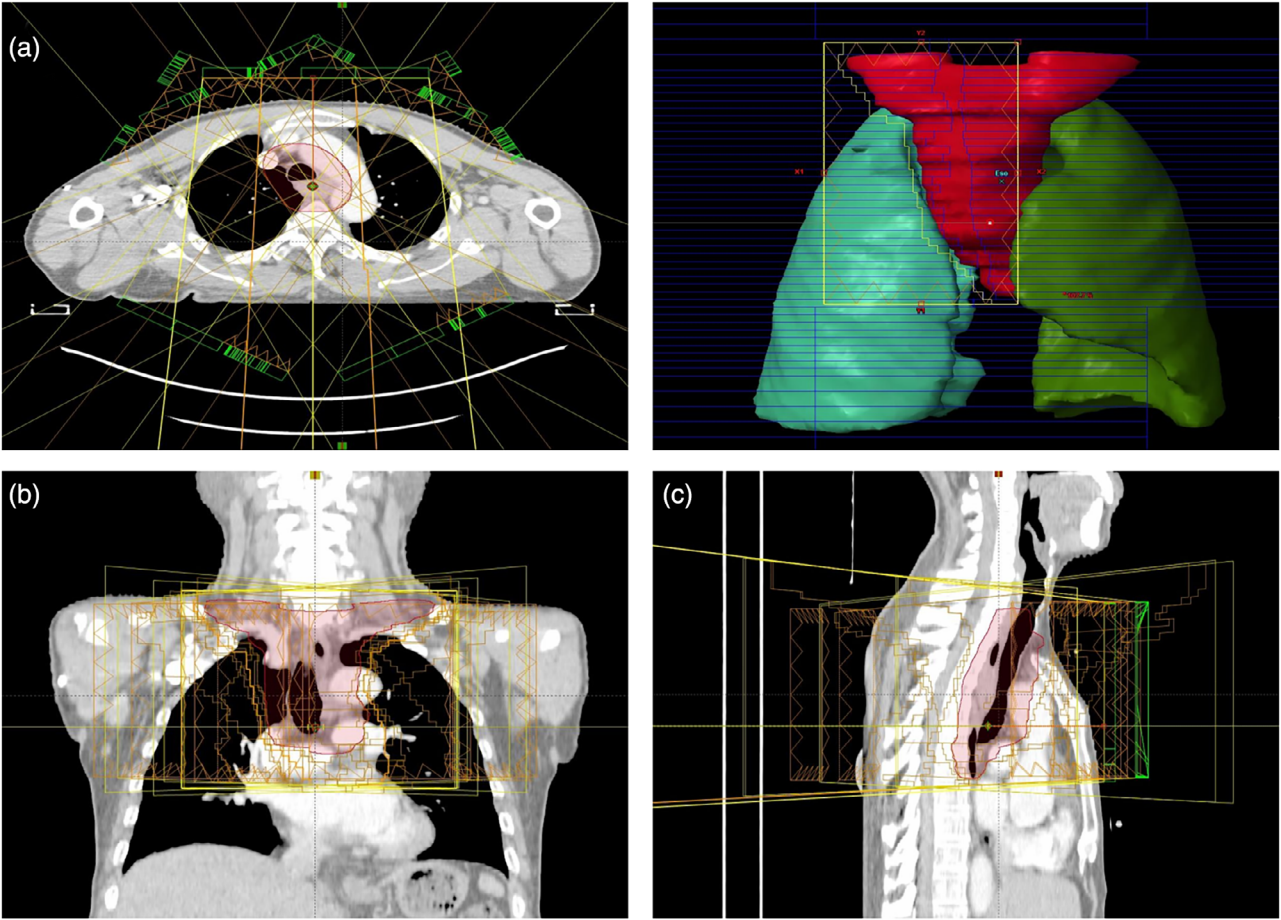
Schematic diagram of EF‐IMRT field for upper esophageal cancer. A: Transverse section, B: Sagittal section, C: coronal section.

The EF‐IMRT technique included elective nodal volumes defined according to international consensus guidelines, encompassing bilateral deep cervical lymphatic chains, supraclavicular regions, and upper mediastinal nodal stations to ensure adequate irradiation of potential subclinical disease. Beam arrangement was performed following the methodology described by Wang et al.,^16^ employing nine coplanar, equally spaced 6‐MV photon beams at gantry angles of 0°, 25°, 50°, 75°, 160°, 200°, 285°, 310°, and 335°. This configuration aimed to minimize beam traversal through lung parenchyma while maintaining robust dose coverage for the prophylactic irradiation target volume for cervical lymph nodes with a large horizontal span. Figure 2 illustrates the beam configuration used in the EF‐IMRT plan: (A) axial view of the seven beams arrangement; (B) coronal view; and (C) sagittal views, demonstrating spatial relationships between beam geometry and the planning target volume.

#### 2.4.2 Segmented Jaw‐locking IMRT (SJL‐IMRT)

Conventional IMRT for upper thoracic esophageal cancer must span a craniocaudal length of ≥20 cm, resulting in extensive MLC motion to accommodate the irregular tumor volume (Shape Complexity Index, SCI).^17^, ^18^ Typically, a more irregular target geometry requires greater MLC motion, higher monitor‐unit (MU) counts, and more complex fluence modulation during IMRT planning. During planning of the EF‐IMRT, the X/Y jaws are set to encompass the required lateral and longitudinal extents (e.g., to match the maximum projection of the target). However, during beam‐on, the collimators remain at the preplanned fixed positions for that beam and do not dynamically follow changes in the MLC aperture on a control‐point basis. Consequently, MLC modulation within the full beam eye view (BEV) of the target could amplify inter‐leaf transmission (>2%), tongue‐and‐groove effects (≈15% underdose),^19^ and leaf‐positioning errors (*σ* = 0.5 mm),^20^ enlarging low‐dose volumes delivered to surrounding organs at risk and compromising peripheral dose conformity.^21^, ^22^ In this paper, we implemented segmented Jaw‐locking IMRT (SJL‐IMRT), a technique employing collimator‐based longitudinal field segmentation. In SJL‐IMRT, the elongated ENI volume is partitioned cranio‐caudally into a cervical–supraclavicular segment (PTV‐C) and an upper‐mediastinal segment (PTV‐T) at the thoracic inlet (sternal notch level, approximately 18 cm from the incisors). Under a single isocenter, we tailor the gantry angle sets for the two longitudinal subfields to reflect their distinct anatomic neighborhoods, with Y‐jaws locked to the corresponding subfield window.

**FIGURE 3:**
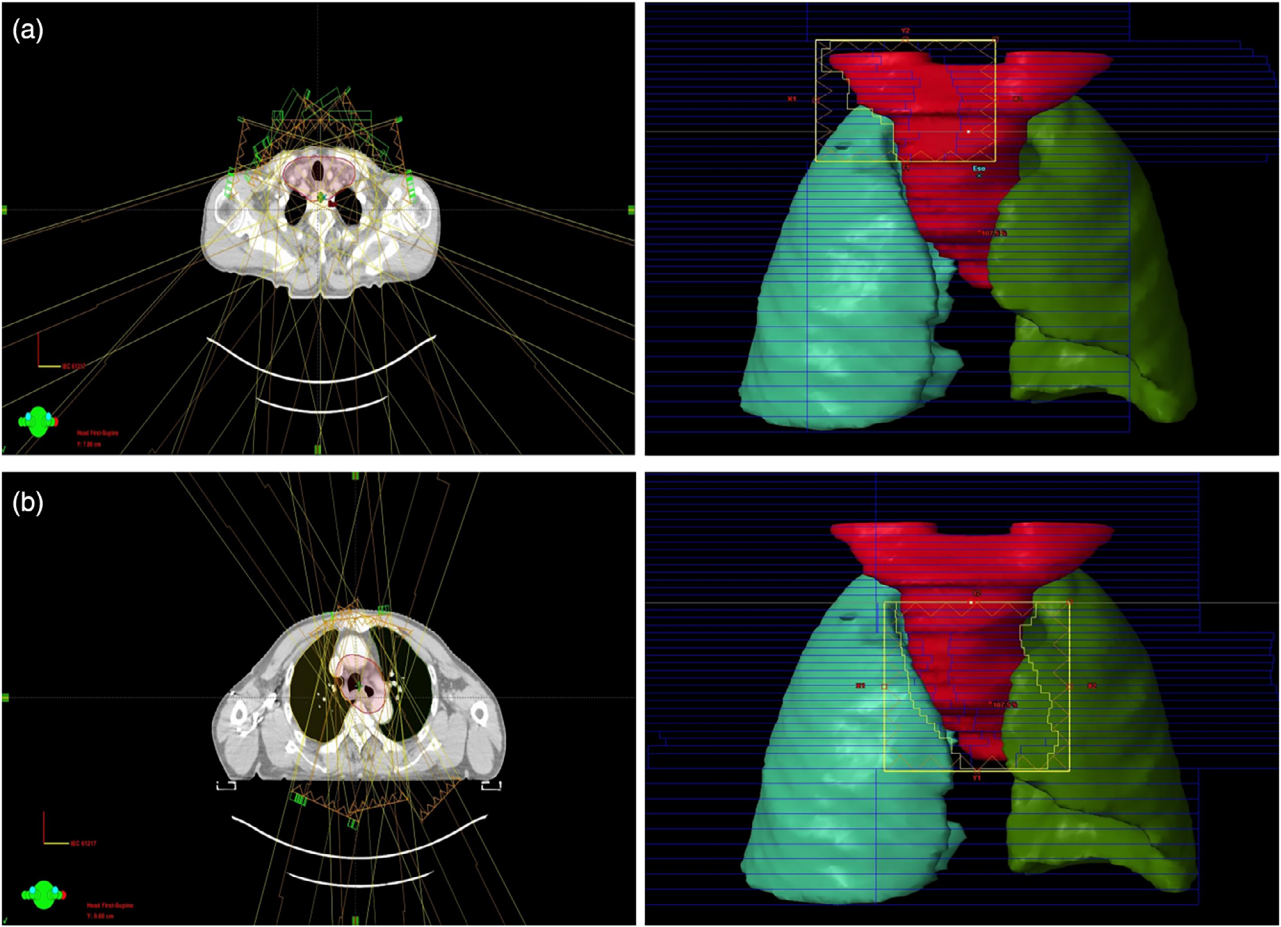
Schematic diagram of SJL‐IMRT field for upper esophageal cancer.

During PTV‐C (cervical–supraclavicular) irradiation (Figure 3a), we intentionally use seven anterior/anterior‐oblique beams (gantry 285°, 310°, 335°, 0°, 25°, 50°, 75°), with PTV‐T physically shielded by orthogonal jaws. The rationale is threefold: (1) “Near‐field” principle for an anterior target. The cervical esophagus lies anteriorly, so anterior/anterior‐oblique incidences shorten the entrance path through non‐target tissues and improve geometric conformity; (2) Spinal canal protection at the beam exit. The spinal canal lies near the distal fall‐off/exit of the beams (posterior to the target), with the vertebral body interposed, thereby reducing integral cord exposure. (3) Minimal lung irradiation in the Cervical Region. In the cervical region, the aerated lung volume intersected by these beams is small. For PTV‐T (upper‐mediastinal) delivery (Figure 3b), we employ a “butterfly” trans‐mediastinal beam set under a single isocenter, with cervical structures excluded by the jaw‐locked window. Specifically, six beams at 330°, 350°, 10°, 30°, 160°, 200° are used to reduce bilateral lung dose—that is, to limit the peripheral low‐dose bath (V_5_) and mean lung dose by avoiding long lateral traversals through lung tissues. It should be clarified that beam count is not intrinsic to the SJL workflow the number of beams can be adapted to patient anatomy or institutional practice.

**FIGURE 4:**
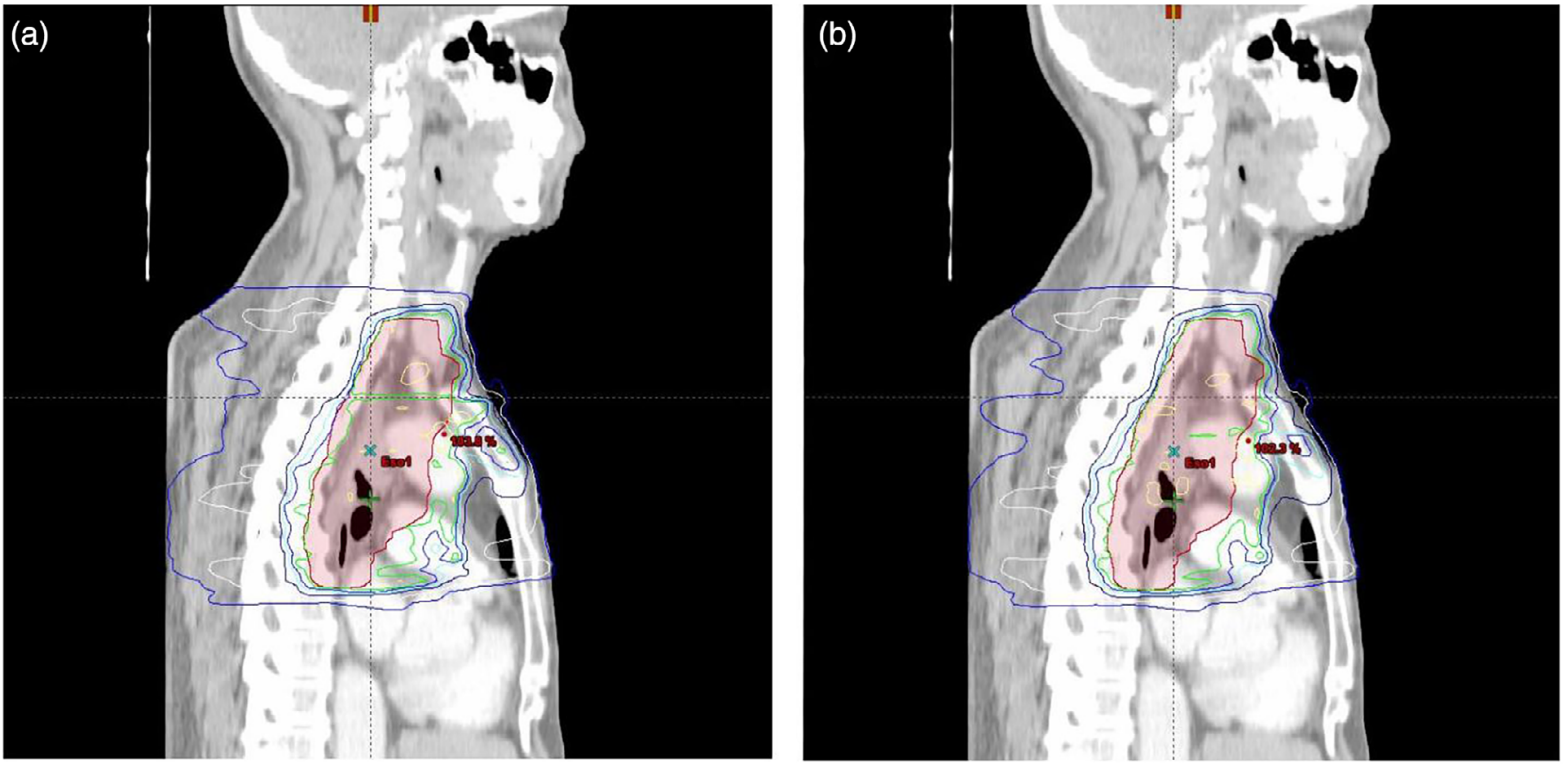
Illustration of optimized dose distribution in the junction area between upper and lower segments of SJL‐IMRT.

Although the segmented independent longitudinal optimization strategy effectively reduces radiation exposure to sensitive tissues, the mechanical boundaries between adjacent longitudinal subfields, particularly at the cervicothoracic junction, can lead to dose discontinuities or inhomogeneities in the junction region (Figure 4a), potentially affecting local tumor control. To address this issue, we introduced an approximately 2 cm longitudinal overlap at the junction of the two jaw‐locked subfields, employing a field feathering strategy^23^ to achieve a smoother dose gradient transition at the junction. In the planning design of the Figure 3, the cranial edge of the PTV‐T subfield window coincides with the isocenter, while the caudal edge of the PTV‐C subfield window extends 2 cm inferior to the isocenter. There is no PTV cropping and no couch shift, the overlap is created entirely at the planning stage. As demonstrated in Figure 4b, this approach effectively mitigated the dose deficit problem at the junction, further enhancing the dosimetric safety and reliability of the SJL‐IMRT technique.

The proposed SJL‐IMRT addressed the limitations of ENI irradiation through a geometric rather than algorithmic intervention, which employs collimator jaw segmentation at the thoracic inlet to divide extended targets into discrete sub‐volumes. MLC modulation is confined to the jaw‐locked windows, while the optimizer iteratively updates beamlet fluence across both cervical and upper thoracic sub‐fields within a single isocenter. This strategy aims to constrain the requisite MLC motion range and modulation complexity, thereby mitigating leakage‐associated dose delivery uncertainties.

### 2.5 Plan evaluation

#### 2.5.1 Plan complexity metrics (EAM and MCS)

To comprehensively evaluate plan feasibility beyond conventional dosimetric parameters, two complementary classes of complexity metrics were introduced: (i) an aperture‐based compactness index, the edge‐area metric (EAM); and (ii) a sequence‐level index, the modulation complexity score (MCS).

EAM is the aperture‐based metric previously quantified the geometric compactness of MLC apertures.^24^, ^25^ The EAM for an individual aperture is mathematically defined as:

(1) $$\mathrm{EA}M_{i}=\frac{P_{i}^{2}}{4\pi A_{i}}$$

where $P_{i}$ represents the aperture perimeter (cm) and $A_{i}$ denotes the aperture area (cm^2^). Consequently, higher EAM values correspond to increased aperture irregularity, characterized by reduced aperture size and highly segmented shapes, thus indicating greater risk in delivery errors. To represent the complexity at the plan level, individual EAM values were weighted by the corresponding fractional monitor units (MU), as given by:

(2) $$\mathrm{EA}M_{\mathrm{plan}}=\sum_{i=1}^{N}\left(\frac{MU_{i}}{\sum_{j=1}^{N}MU_{j}}\right)EAM_{i}$$

where *N* denotes the total number of apertures in the treatment plan. Given that SJL‐IMRT adopts longitudinal Jaw‐locking segmentation, which inherently reduces target elongation and irregularities, this study separately computed $\mathrm{EA}M_{\mathrm{plan}}$ values for cervical/supraclavicular (PTV‐C) and upper thoracic segments (PTV‐T). Furthermore, correlations between the plan‐level complexity and critical dosimetric endpoints, including lung low‐dose exposure parameters (V_5_, mean lung dose), were analyzed to elucidate potential clinical and quality assurance advantages conferred by the geometric simplification of SJL‐IMRT.

While aperture‐based indices focus on geometric properties, MCS quantifies the variability of dynamic MLC sequencing during plan delivery. MCS is defined as the product of aperture area variability (AAV) and leaf sequence variability (LSV), averaged over adjacent control point intervals and weighted by monitor units (MU)^26^, ^27^:

(3) $$\mathrm{MCS}=\frac{\sum_{i}MU_{i}\cdot \left(\frac{AAV_{i-1}+AAV_{i}}{2}\cdot \frac{LSV_{i-1}+LSV_{i}}{2}\right)}{\sum_{i}MU_{i}}$$

where AAV describes the relative aperture area compared with the maximum opening of the beam, with lower values indicating larger variability and higher modulation. LSV reflects the smoothness of leaf sequencing, with smaller values corresponding to more irregular leaf arrangements. MCS values range from 0 to 1, where higher scores represent simpler, more deliverable plans, while lower scores indicate greater modulation complexity.

Together, EAM and MCS provide two orthogonal perspectives on plan complexity: the former captures geometric compactness of apertures, whereas the latter characterizes dynamic sequencing complexity of MLC movements. Using both metrics enables a more comprehensive evaluation of plan deliverability and modulation.

#### 2.5.2 Dosimetric metrics

Dose‐volume histograms (DVHs) were employed to evaluate dose distributions for the target volumes and organs at risk (OARs). Evaluation parameters included near‐maximum dose (D_2%_), near‐minimum dose (D_98%_), conformity index (CI), homogeneity index (HI),^28^ and gradient measure (GM),^29^ defined as follows:

D_2%_: Dose received by at least 2% of the target volume, representing high‐dose hotspots.
D_98%_: Dose received by at least 98% of the target volume, representing low‐dose cold spots.
CI: Measures how well the prescribed dose conforms to the target volume, calculated as:
(4) $$\mathrm{CI}=\frac{V_{RI}}{V_{PTV}}$$
  where $V_{RI}$ represents the volume covered by the prescribed dose, and $V_{PTV}$ is the planning target volume. A CI closer to 1 indicates superior conformity.
HI: Describes dose uniformity within the target, defined as:
(5) $$\mathrm{HI}=\frac{D_{2\%}-D_{98\%}}{D_{p}}$$
  where represents the prescribed dose. An HI approaching 0 indicates more homogeneous dose distribution.
GM [cm]: The maximum radial distance from the PTV edge to the point receiving 50% of the prescription dose. GM evaluates the dose gradient, with smaller values indicating steeper gradients and improved protection of adjacent normal tissues.
(6) $$\mathrm{GM}=\max\left(D_{50\%}^{\mathrm{surface}}\right)-R_{\mathrm{PTV}}$$

Here, $D_{50\%}^{\mathrm{surface}}$ represents the maximum distance from the PTV boundary to the 50% prescription isodose surface, and $R_{PTV}$ denotes the geometric equivalent radius of the target, calculated from the target volume ($V_{PTV}$) as:

(7) $$R_{\mathrm{PTV}}={\left(\frac{3V_{\mathrm{PTV}}}{4\pi }\right)}^{1/3}$$

Additionally, dosimetric parameters employed to assess the sparing of organs at risk (OARs) included mean lung dose (MLD) and lung volume percentages receiving at least 5, 10, 13, 15, 20, and 30 Gy (V_5_, V_10_, V_13_, V_15_, V_20_, V_30_), spinal cord maximum (D_max_).

#### 2.5.3 Biological metrics (TCP and NTCP)

Biological metrics, including tumor control probability (TCP) and normal tissue complication probability (NTCP), were additionally employed for plan evaluation. Compared with traditional dosimetric indices (CI, HI), TCP and NTCP provide a direct quantification of the biological impact of varying dose distributions on tumor cell eradication and normal tissue injury, thereby offering quantitative guidance for clinical decision‐making.^30^ The TCP was calculated based on the equivalent uniform dose (EUD) model:^31^

(8) $$\mathrm{TCP}=\frac{1}{1+{\left(\frac{TCD_{50}}{\mathrm{EUD}}\right)}^{4\gamma_{50}}}$$

(9) $$\mathrm{EUD}={\left(\sum_{i=1}^{N}v_{i}\;D_{i}^{a}\right)}^{\frac{1}{a}}$$

where $TCD_{50}$ represents the dose corresponding to a 50% tumor control probability, and $\gamma_{50}$ denotes the slope of the TCP curve at the 50% probability point. Additionally, $v_{i}$ indicates the relative volume of the i‐th voxel, $D_{i}$ is the dose delivered to the i‐th voxel, and a characterizes the dose sensitivity of the tissue. According to the study by Okunieff et al.,^32^ the TCP model of esophageal cancer has a value of $TCD_{50}$ = 49.09 Gy, $\gamma_{50}$ = 2.16, and a = −8. Normal tissue complication probability (NTCP) is widely applied to evaluate the risk of complications in organs at risk (OARs) receiving inhomogeneous radiation doses.^33^ NTCP is calculated as follows:

(10) $$\mathrm{NTCP}=\frac{1}{1+{\left(\frac{TD_{50}}{\mathrm{EUD}}\right)}^{4\gamma_{50}}}$$

where $TD_{50}$ denotes the dose corresponding to a 50% complication rate, EUD represents the equivalent uniform dose, and $\gamma_{50}$ indicates the slope of the NTCP dose‐response curve at the 50% probability point. Table 2 summarizes the parameters used for calculating TCP for esophageal cancer and NTCP values for critical organs (lung and spinal cord) in this study.^34^

**TABLE 2:** Parameters used for the calculation of the TCP and NTCP.

	$TCD_{50}$ (Gy)	$TD_{50}$ (Gy)	a	$\gamma_{50}$
TCP	49.09	–	−8	2.16
NTCP lung	–	34	3	0.9
NTCP spinal core	–	68.6	13	1.9

### 2.6 Plan verification

Plan verification for the SJL‐IMRT and EF‐IMRT plans was performed with the two‐dimensional ionization chamber array (PTW OCTAVIUS 729 2D Array, 729 chambers in 27×27 matrix, sensitive volume 0.5 cm^3^ per chamber; PTW‐Freiburg, Germany) embedded in the RW3 Slab Phantom (Type T29672; PTW‐Freiburg, Germany). The detector's effective measurement plane was positioned at 5.0 cm depth beneath the incident surface, with 8.0 cm of solid water providing backscatter below the detector plane. Verification plans were generated by transferring each treatment field from the original patient plans to the phantom geometry in the Varian Eclipse TPS (Version 13.1), with all gantry angles fixed at 0° while preserving identical beam parameters. Fixed 0° gantry angle verification is recommended for eliminating mechanical uncertainties from gantry rotation and focusing on dose calculation and MLC accuracy assessment.^35^ Measured dose distributions were compared to TPS calculations using gamma analysis (3.0%/3.0 mm criterion, global normalization, 10% maximum dose threshold), *γ* passing rate (3 mm/3%) ≥95% is considered as the acceptable standard for dose calculation and MLC accuracy following AAPM tg‐218 recommendations.^36^

### 2.7 Statistical analysis

Statistical analyses were conducted using SPSS Statistics (version 22.0). Paired Wilcoxon signed‐rank test^37^ was performed for within‐subject comparisons between groups. Differences were considered statistically significant if the two‐tailed *p*‐value was less than 0.01.

## 3 RESULTS

### 3.1 Dose distribution comparison of plans

**FIGURE 5:**
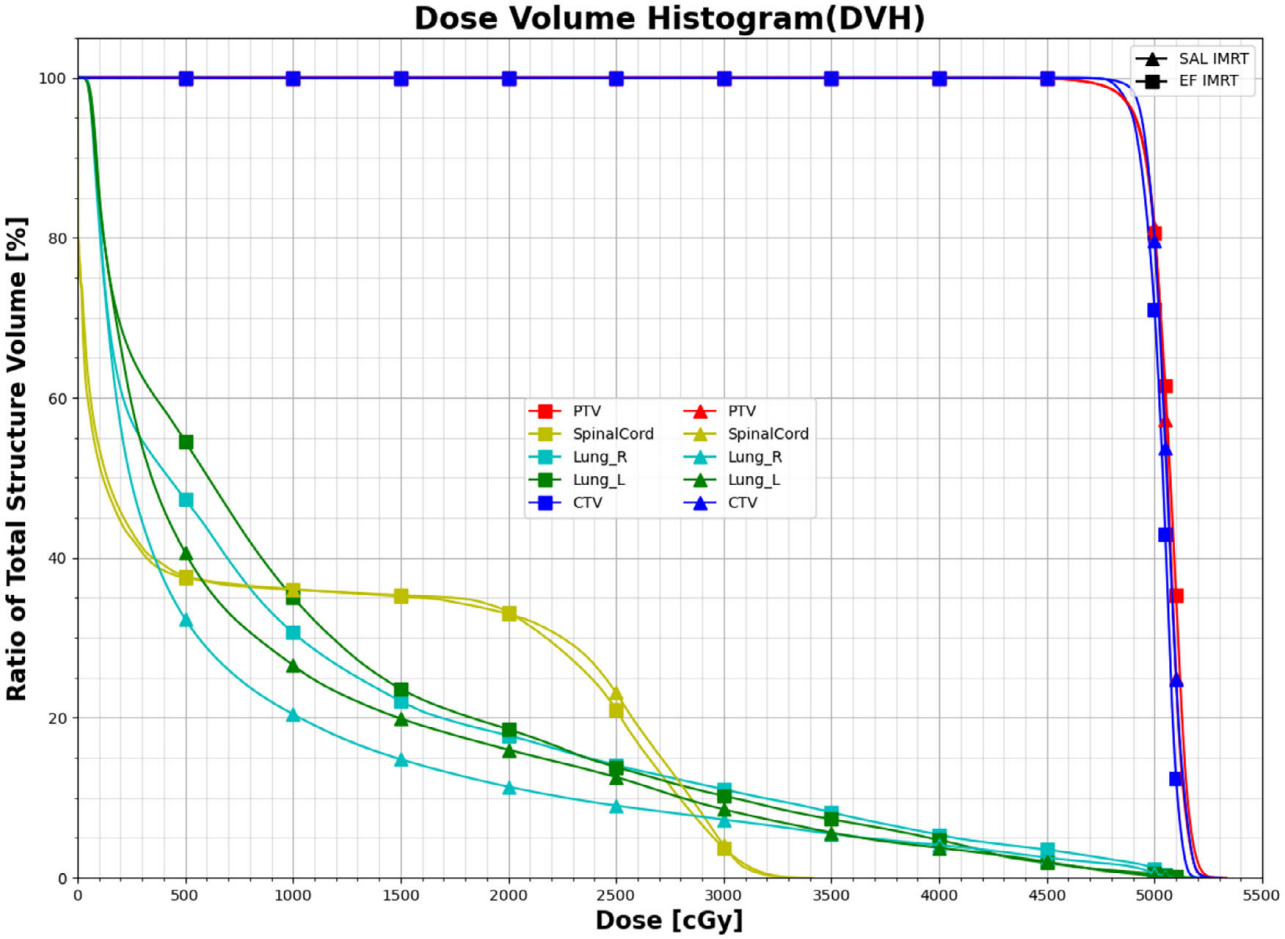
DVH for the target and organs at risks of the same patient.

**FIGURE 6:**
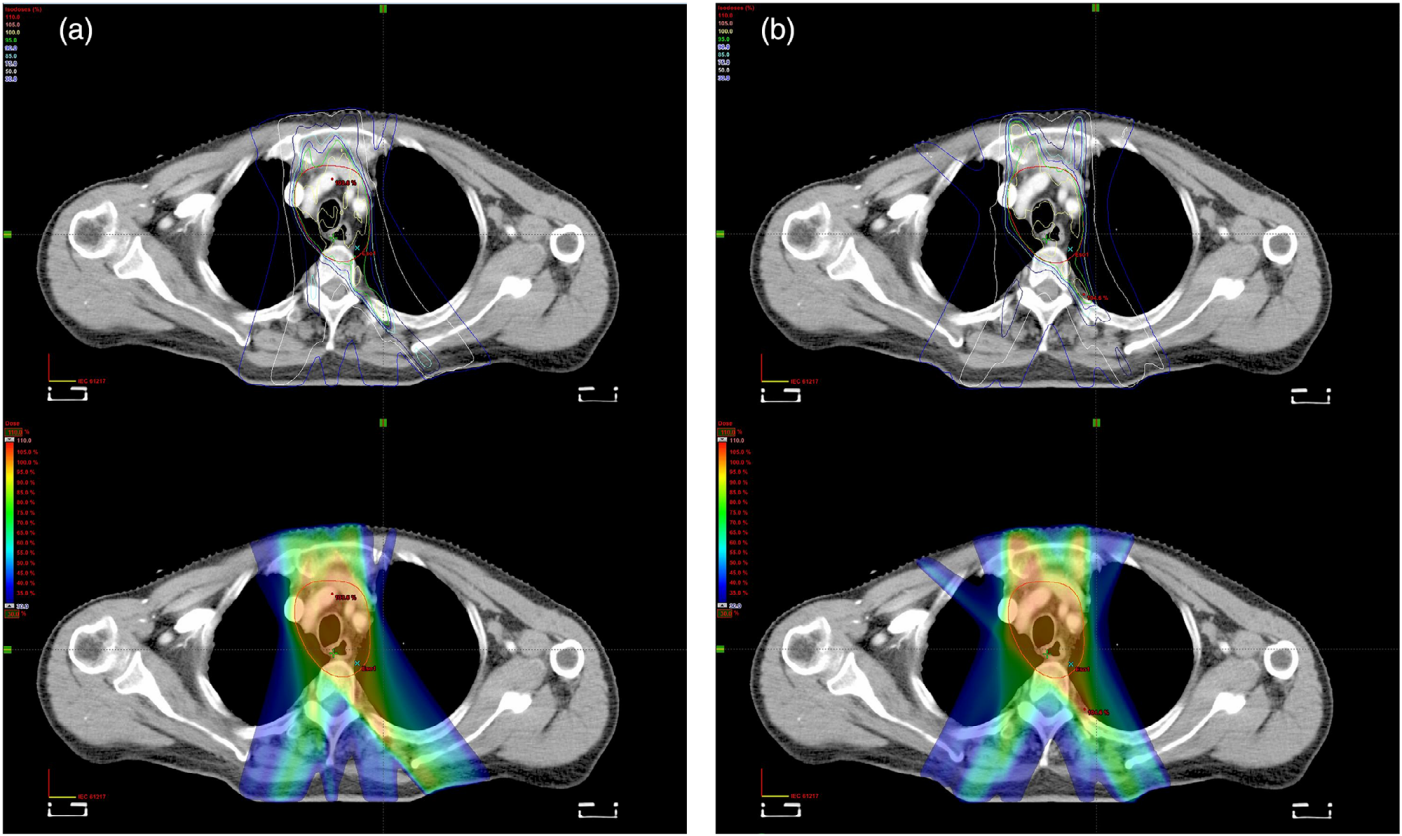
Planned isodose distribution for the same patient.

All SJL‐IMRT and EF‐IMRT plans satisfied predefined clinical dose constraints. Figure 5 compares dose‐volume histograms (DVHs) for a representative case, demonstrating that SJL‐IMRT significantly reduced lung exposure at intermediate‐to‐low dose levels while maintaining comparable target coverage. Figure 6 illustrates axial dose distributions for the same patient, with peripheral blue contours representing the 30% isodose lines and peripheral white contours indicating the 50% isodose lines. The SJL‐IMRT plan (Figure 6a) exhibited tighter dose distributions around the PTV boundary, demonstrating steeper dose gradients and notably smaller lung volumes exposed to low‐dose radiation compared with EF‐IMRT (Figure 6b).

### 3.2 Complexity comparison of plans

Table 3 summarizes the paired comparison of plan‐level complexity between SJL‐IMRT and EF‐IMRT. After MU‐weighted pooling of all segmented Jaw‐locking beams across the cervical and thoracic segments to form a single plan metric, SJL‐IMRT exhibited a substantially lower EAM than EF‐IMRT (8.610 ± 2.951 vs. 20.824 ± 4.944), with a mean paired difference of −12.214 and a Wilcoxon signed‐rank test *p* = 0.00195. In contrast, the MCS was higher for SJL‐IMRT than for EF‐IMRT (0.243 ± 0.015 vs. 0.203 ± 0.036), with a mean paired difference of 0.040 and *p* = 0.0193. These two metrics indicate that SJL‐IMRT decreases geometric aperture complexity while improving deliverability of the MLC sequence.

**TABLE 3:** Comparisons of plan complexity (mean ± std).

Parameter	SJL‐IMRT	EF‐IMRT	Difference	*p* value
EAM	8.610 ± 2.951	20.824 ± 4.944	−12.214 ± −1.993	0.00195
MCS	0.243 ± 0.015	0.203 ± 0.036	0.04 ± −0.021	0.0193

### 3.3 Dosimetric comparison of target volumes

Table 4 compares dosimetric parameters for PTV between SJL‐IMRT and EF‐IMRT plans (mean ± std), with all data presented as mean ± standard deviation. No statistically significant differences were observed in near‐maximum dose (D_2%_: 51.79 ± 0.23 Gy vs. 51.70 ± 0.39 Gy, *p* = 0.071), near‐minimum dose (D_98%_: 48.62 ± 0.53 Gy vs. 48.68 ± 0.60 Gy, *p* = 0.047), conformity index (CI: 0.973 ± 0.103 vs. 0.962 ± 0.188, *p* = 0.058) and homogeneity index (HI: 0.075 ± 0.011 vs. 0.081 ± 0.008, *p* = 0.039). However, SJL‐IMRT significantly improved dose gradient steepness, with gradient measure (GM) decreasing from 4.354 ± 0.612 cm to 4.139 ± 0.658 cm (Δ = −0.215 cm, *p*<0.01), equivalent to a 4.9% reduction in gradient distance. This enhancement indicates SJL‐IMRT's superior capability to confine 50% dose region adjacent to lung tissue, consistent with the contracted 50% isodose lines (white contours) at the PTV‐lung interface in Figure 4.

**TABLE 4:** Comparisons of PTV dosimetric parameters (mean ± std).

Parameter	SJL‐IMRT	EF‐IMRT	Difference	*p* value
SCI				
D_2%_ (Gy)	51.79 ± 0.23	51.70 ± 0.39	0.09	0.071
D_98%_ (Gy)	48.62 ± 0.53	48.68 ± 0.60	−0.06	0.047
CI	0.973 ± 0.103	0.962 ± 0.188	0.011	0.058
HI	0.075 ± 0.011	0.081 ± 0.008	−0.006	0.039
GM (cm)	4.139 ± 0.658	4.354 ± 0.612	−0.215	<0.01

### 3.4 Dosimetric comparison of OARs

Table 5 summarizes dosimetric parameters for organs at risk (OARs) across all patients, corresponding to the DVH in Figure 3. Compared with EF‐IMRT, SJL‐IMRT demonstrated statistically significant reductions in all lung dose‐volume parameters, with left lung V_5_ decreasing from 73.61 ± 1.42% to 53.65 ± 8.96% (27.1% relative reduction) and right lung V_5_ from 71.48 ± 18.29% to 46.21 ± 10.09% (35.4% relative reduction), while mean lung dose (MLD) decreased by 1.62 Gy (left) and 2.64 Gy (right) (*p *< 0.01). Both techniques maintained spinal cord D_max_ below the 35 Gy clinical tolerance threshold (SJL: 33.99 ± 0.86 Gy vs. EF: 34.14 ± 0.89 Gy, *p* = 0.023), confirming SJL‐IMRT's superior normal tissue sparing capability within clinically acceptable constraints. Moreover, significant improvements were consistently observed in intermediate‐dose regions (V_10_–V_20_). For the left lung, SJL‐IMRT reduced V_10_, V_13_, V_15_, and V_20_ by 5.47%, 3.48%, 3.18%, and 3.63%, respectively (all *p *< 0.01). Similarly, for the right lung, reductions of 9.67% (V_10_), 5.02% (V_13_), 3.76% (V_15_), and 3.05% (V_20_) were achieved, demonstrating SJL‐IMRT's consistent advantage across clinically relevant dose ranges. Although V_30_ values for both lungs were within acceptable clinical thresholds, SJL‐IMRT further reduced left and right lung V_30_ by 1.54% (*p* = 0.0402) and 1.85% (*p* = 0.0247), respectively, further highlighting the dosimetric benefits of this approach.

**TABLE 5:** Comparison of the OARs dosimetric parameters of the two techniques.

OARs	SJL‐IMRT	EF‐IMRT	Difference	*p* value
Left lung				
MLD (Gy)	11.26 ± 1.84	12.88 ± 1.23	−1.62	<0.01
V_5_ (%)	53.65 ± 8.96	73.61 ± 1.42	−19.96	<0.01
V_10_ (%)	34.41 ± 5.66	39.88 ± 3.12	−5.47	<0.01
V_13_ (%)	27.81 ± 4.37	31.29 ± 2.85	−3.48	<0.01
V_15_ (%)	24.79 ± 4.07	27.97 ± 2.81	−3.18	<0.01
V_20_ (%)	19.03 ± 3.86	22.66 ± 2.59	−3.63	<0.01
V_30_ (%)	10.58 ± 2.68	12.12 ± 2.68	−1.54	0.0402
Right lung				
MLD (Gy)	9.53 ± 1.84	12.17 ± 1.92	−2.64	<0.01
V_5_ (%)	46.21 ± 10.09	71.48 ± 18.29	−25.27	<0.01
V_10_ (%)	28.74 ± 6.06	38.41 ± 7.24	−9.67	<0.01
V_13_ (%)	23.57 ± 5.03	28.59 ± 4.94	−5.02	<0.01
V_15_ (%)	21.02 ± 4.53	24.78 ± 4.41	−3.76	<0.01
V_20_ (%)	15.89 ± 3.90	18.94 ± 3.84	−3.05	<0.01
V_30_ (%)	7.64 ± 2.89	9.49 ± 3.22	−1.85	0.0247
Spinal cord				
D_max_ (Gy)	33.99 ± 0.86	34.14 ± 0.89	−0.15	0.023

### 3.5 Comparison of delivery validation

**FIGURE 7:**
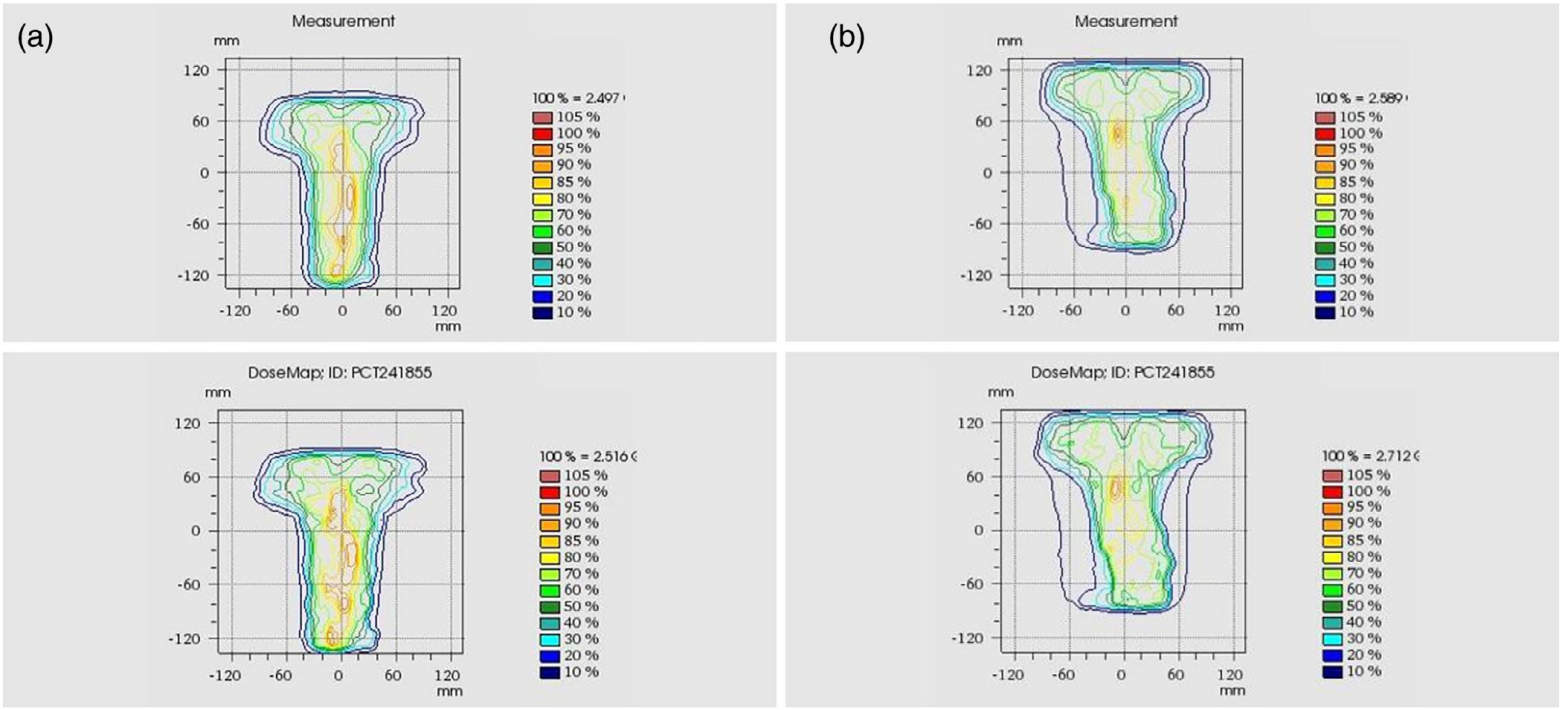
QA plan dose distribution map. (a) SJL‐IMRT, (b) EF‐IMRT.

The delivery accuracy of the EF‐IMRT and SJL‐IMRT plans was validated through assurance (QA), and compared to TPS‐calculated dose maps using gamma analysis (3.0%/3.0 mm criterion, global normalization, 10% dose threshold). Figure 7 illustrates measured dose distributions (upper row) and the corresponding TPS‐calculated map (lower row) for representative SJL‐IMRT and EF‐IMRT OA deliveries. SJL‐IMRT measurements exhibit no detectable dose cold or hotspot at the 2‐cm feathered overlap region, and isodose lines across the junction display a smooth and continuous gradient transition. Moreover, compared with EF‐IMRT, SJL‐IMRT presents a more compact and conformal high‐dose region, implying a reduced risk of incidental exposure to adjacent organs. This can be attributed to the proposed segmentation strategy constrains tongue‐and‐groove effects and requisite leaf transmission to mitigate leakage‐related uncertainties in dose delivery. Furthermore, the reduction in EAM‐based geometric irregularity and the smoother sequencing reflected by higher MCS supports the aforementioned dosimetric benefits from the perspective of plan complexity.

The deliverability of SJL‐IMRT and EF‐IMRT was evaluated using *γ* analysis and delivery metrics. As summarized in Table 6, SJL‐IMRT achieved a marginally higher mean γ‐passing rate than EF‐IMRT (99.4 ± 0.72% vs. 98.8 ± 0.75%; *p* = 0.048), indicating good agreement between measured and calculated dose distributions for both techniques. The average delivery time was longer for SJL‐IMRT (10.9 vs. 8.8 min; Δ = 2.1 min, *p* = 0.021) and SJL‐IMRT exhibited a higher total MU (2893 vs. 2066; Δ = 827, *p* = 0.026). The segmented field geometry intrinsically increases the number of independent beam segments that must deliver dose within their restricted longitudinal apertures. This results in a higher delivery time and cumulative MU, even though the modulation within each segment is simpler.

**TABLE 6:** Comparisons of plan deliverability parameters.

Plan type	Mean γ passing rate (%)	Mean delivery time (min)	Mean MUs
SJL‐IMRT	99.4 ± 0.72	10.9	2893
EF‐IMRT	98.8 ± 0.75	8.8	2066
Difference	0.6 ± −0.03	2.1	827
*p* value	0.048	0.021	0.026

### 3.6 Comparison of tumor control probability (TCP) and normal tissue complication probability (NTCP)

Table 7 summarizes the comparison of tumor control probability (TCP) for the target volumes (PTV and CTV) and normal tissue complication probability (NTCP) for lungs and spinal cord, calculated using MATLAB R2024a. Although SJL‐IMRT showed modest increases in TCP for both the PTV (0.67%; *p* = 0.029) and CTV (0.51%, *p* = 0.038) compared to EF‐IMRT, these differences did not reach high statistical significance (*p*>0.01). Nonetheless, the observed numerical improvements suggest a potential clinical advantage in tumor control attributable to the optimized segmented dose distribution. Conversely, SJL‐IMRT significantly reduced the NTCP values for bilateral lungs, achieving an average reduction of 1.30% (*p*<0.01) for the left lung and 1.29% (*p*<0.01) for the right lung. These reductions highlight SJL‐IMRT's enhanced capability to minimize lung toxicity, potentially translating to reduced clinical incidence of radiation pneumonitis. No statistically significant difference was observed in NTCP for spinal cord between SJL‐IMRT and EF‐IMRT (0.27% vs. 0.41%; difference: −0.14%; *p* = 0.036), the slight numerical reduction indicates SJL‐IMRT may confer additional safety margins within clinically acceptable constrains. Collectively, these findings underscore the biological and clinical advantages of SJL‐IMRT, particularly in mitigating pulmonary complications while maintaining, if not slightly enhancing, tumor control.

**TABLE 7:** Comparisons of TCP and NTCP (mean ± std).

	SJL‐IMRT	EF‐IMRT	Difference	*p* value
$\mathrm{TC}P_{PTV}$ (%)	56.82 ± 2.51	56.15 ± 2.11	0.67	0.029
$\mathrm{TC}P_{CTV}$ (%)	59.30 ± 4.08	58.79 ± 3.47	0.51	0.038
$\mathrm{NTC}P_{left\_lung}$ (%)	7.07 ± 2.67	8.37 ± 2.90	−1.3	<0.01
${NTCP}_{right\_lung}$ (%)	5.78 ± 2.54	7.07 ± 2.93	−1.29	<0.01
$\mathrm{NTC}P_{spinal\_cord}$ (%)	0.27 ± 0.26	0.41 ± 0.23	−0.14	0.036

## 4 DISCUSSION

In upper thoracic esophageal cancer, the elective nodal irradiation (ENI) volume is both elongated and highly concave, imposing substantial shape complexity on the target. Under conventional IMRT, the MLC must perform wide‐range, finely modulation to conform dose to this irregular geometry, which magnifies inter‐leaf transmission, tongue‐and‐groove effects, and leaf positioning uncertainty. The downstream consequence is a broader intermediate‐to‐low‐dose radiation exposure to adjacent normal tissues, particularly the lungs and spinal cord, precisely the pattern most strongly correlated with clinically significant radiation pneumonitis and other toxicities.^38^ To balance therapeutic efficacy and protection of normal tissues, this study employed a segmented Jaw‐locking IMRT (SJL‐IMRT) strategy that independently optimizes the cervical (supraclavicular) and upper thoracic (upper mediastinal) regions of the esophageal target volume via locking the jaw‐defined aperture longitudinally within each segment. Conceptually, aperture locking acts as a geometric regularizer by limiting the active longitudinal span and suppressing unnecessary open and internal notches, it is expected to reduce plan complexity (i.e., larger, more uniform effective apertures and smoother leaf trajectories, reflected by lower aperture‐based complexity and higher sequence smoothness), which in turn should decrease intermediate‐to‐low‐dose (e.g., reduced low‐dose spillage and shorter fluence tails) and thereby lower lung NTCP under EUD model‐based formalisms.

Table 3 may elucidate mechanism underlying for the dosimetric advantage of segmented aperture locking. The aperture‐based complexity metric (EAM) was substantially lower with SJL‐IMRT than with EF‐IMRT (8.610 ± 2.951 vs. 20.824 ± 4.944; paired mean difference Δ = −12.214 ± 1.993; *p* = 0.00195). Given EAM is defined as $P^{2}/\left(4\pi A\right)$, the reduction in EAM indicates that, under segmentation, aperture area (*A*) increases disproportionately relative to perimeter (*P*). Jaw‐based aperture locking restricts the active longitudinal span and suppresses non‐contributory open rows and internal notches. In other words, segmentation regularizes the beam shape, yielding larger, more compact, and less serrated apertures at each control‐point interval. MU‐weighted pooling then emphasizes these simpler intervals at the plan level. Table 3 further shows that SJL‐IMRT achieves a higher modulation complexity score (MCS) than EF‐IMRT (0.243 ± 0.015 vs. 0.203 ± 0.036; paired difference Δ = 0.04 ± −0.021; *p* = 0.0193), indicating simpler, more deliverable leaf sequencing. Mechanistically, MCS is the MU‐weighted average of the product of AAV and LSV, AAV (aperture area variability) measures relative aperture occupancy, and LSV (leaf sequence variability) quantifies how smoothly neighboring leaves line up within each bank. Both range from 0 to 1, where larger values indicate more open apertures and smoother, less jagged profiles, respectively. In SJL‐IMRT, by constraining the longitudinal modulation window, jaw‐based segmentation: (i) widens effective leaf gaps in dose‐contributing rows (raising AAV toward the beam's achievable maximum) and (ii) reduces abrupt neighboring‐leaf differences and transient narrow gaps (raising LSV). MU weighting then emphasizes these better‐occupied, smoother intervals, yielding an overall more deliverable plan. Together, the EAM↓ and MCS↑ findings support the proposed mechanism: aperture locking lowers geometric and sequential complexity, which is expected to shorten longitudinal fluence tails, a pathway consistent with reductions in the intermediate‐to‐low‐dose bath to off‐target lung parenchyma.

As shown in Table 4, dosimetric comparison between the proposed segmented Jaw‐locking IMRT (SJL‐IMRT) and conventional extended‐field IMRT (EF‐IMRT) indicated no statistically significant differences (*p *> 0.01) regarding target coverage metrics, including near‐maximum dose (D_2%_), near‐minimum dose (D_98%_), conformity index (CI), and homogeneity index (HI). This result demonstrates that both techniques effectively meet clinical requirements for target conformity and dose homogeneity. However, SJL‐IMRT provided a modest increase in D_98%_ by 0.34 Gy, indicating slightly improved coverage at the low‐dose edge of the target volume. Additionally, the gradient measure (GM) was significantly reduced by 0.215 cm with SJL‐IMRT, indicating a steeper dose fall‐off outside the target, thereby effectively reducing radiation exposure to adjacent normal tissues, which is consistent with findings presented in Table 5.

Radiation pneumonitis (RP) remains one of the most clinically relevant toxicities in thoracic radiotherapy for esophageal cancer. NRG Oncology/RTOG 1010 trial and NCCN Clinical Practice Guidelines in Oncology (Version 4.2025)^39^, ^40^ emphasize that dose‐volume metrics and mean lung dose (MLD) are well‐validated predictors of ≥grade 2 RP, particularly in patients undergoing concurrent chemoradiotherapy. The guidelines recommend limiting V_5_ to 55%, V_20_ to 25% and mean lung dose (MLD) to 13 Gy or less. Table 4 systematically illustrates that SJL‐IMRT consistently met key lung dose constraints across our cohort, with notable advantages in low‐dose metrics. Specifically, left lung V_5_ was 53.65% and right lung V_5_ was 46.21%, which represented the most pronounced dosimetric benefit of this technique. In contrast, EF‐IMRT failed to meet guideline constraints for bilateral lung V_5_ (left lung: 73.61%, right lung: 71.48%), exceeding the RTOG‐recommended threshold of <55%. For V_20_ and mean lung dose (another two critical predictors of radiation pneumonitis), SJL‐IMRT further demonstrated superior compliance with guidelines: V_20_ was 19.03% (left lung) and 15.89% (right lung), while MLD was 11.26 Gy (left lung) and 9.53 Gy (right lung). Although EF‐IMRT marginally met the NCCN V_20_ and MLD threshold (left lung V_20_: 22.66%, MLD: 12.88 Gy; right lung V_20_: 18.94%, MLD: 12.17 Gy), its values were substantially higher than those of SJL‐IMRT. Given the well‐established dose‐response relationship between lung dose metrics and radiation pneumonitis risk, EF‐IMRT exhibited inferior robustness in mitigating radiation pneumonitis risk compared to SJL‐IMRT. Furthermore, although the absolute values of lung V_30_ in both techniques remained well within the acceptable RP clinical threshold of 18% proposed by Inoo et al.,^41^ the additional reductions achieved with SJL‐IMRT provide further safety margins (1.54% reduction in left lung and 1.85% reduction in right lung), potentially enhancing patient tolerance and reducing the long‐term incidence of radiation‐induced lung injury.

As summarized in Table 6, across 40 paired deliveries, SJL‐IMRT exhibited a 2.1‐min increase in mean delivery time, consistent with the modest overhead introduced by longitudinal segmentation but partially mitigated by smoother leaf sequencing. Importantly, this increase in treatment time did not compromise delivery accuracy, where SJL‐IMRT achieved a slightly higher mean γ‐passing rate than EF‐IMRT, indicating that the mechanically simpler apertures and reduced unnecessary modulation supported more stable beam delivery. The segmented geometry inherently necessitates additional beam segments, which accounts for the higher total monitor units. Specifically, the increased total MU observed in SJL‐IMRT is a direct geometric consequence of longitudinal segmentation, rather than an indication of elevated modulation complexity. Since each sub‐aperture irradiates only a portion of the PTV per time, the optimizer compensates for the reduced effective aperture area by assigning proportionally higher beam intensity to individual segments to achieve uniform target coverage. Consequently, the cumulative MU increases despite simpler modulation within each segment. Collectively, dosimetric and mechanical data indicate that the prolongation in delivery time and increase in MUs represent an acceptable trade‐off for the substantial reductions in intermediate‐to‐low lung dose and enhanced delivery robustness conferred by SJL‐IMRT.

This study further evaluated tumor control probability (TCP) and normal tissue complication probability (NTCP) to validate the potential biological advantages of SJL‐IMRT. As illustrated in Table 7, SJL‐IMRT significantly reduced low‐dose radiation exposure (V_5_, V_20_) to lung tissues, thereby achieving notable reductions in lung NTCP values (left lung: decreased by 1.30%; right lung: decreased by 1.29%; both *p *< 0.01). Additionally, spinal cord NTCP was reduced by 0.14%. Considering the direct association between NTCP and clinical radiation‐induced complication rates, these findings suggest that SJL‐IMRT might clinically benefit patients by lowering the incidence of radiation pneumonitis and spinal cord injury. However, it is important to note that despite promising dosimetric and biological indicators suggesting reduced risks of RP and other complications, further prospective clinical studies are necessary to confirm these benefits. Although TCP values for PTV and CTV in SJL‐IMRT showed no statistically significant improvement compared to EF‐IMRT, SJL‐IMRT still demonstrated numerically superior outcomes (0.67% increase for PTV and 0.51% increase for CTV). This advantage could be attributed to reduced low‐dose exposure to surrounding normal tissues, thus achieving a better clinical balance between effective tumor control and normal tissue sparing.

## 5 CONCLUSION

In summary, this study proposed and evaluated a segmented Jaw‐locking IMRT (SJL‐IMRT) strategy to mitigate excessive OARs exposure during elective nodal irradiation (ENI) for upper thoracic esophageal cancer. By partitioning a highly modulated long field into two lower complexity segments, SJL‐IMRT reduced dosimetric uncertainties associated with extensive longitudinal modulation and achieved clinically meaningful OAR sparing without compromising target homogeneity or delivery robustness. Notably, 4D‐CT simulation is the standard of care for thoracic malignancies to account for respiratory‐induced target motion, our study did not integrate 4D‐CT‐based internal target volume (ITV) delineation or adaptive radiotherapy strategies. Future prospective investigations of SJL‐IMRT should incorporate these components as standard workflow elements to further evaluate the long‐term biological benefits of organ sparing.

## AUTHOR CONTRIBUTIONS

All authors were involved in preparation of the manuscript. Shi Cao and Guang‐Zhi Sun designed and performed the research, and wrote the paper. Yi‐Hai Fang and Wan‐Song Xu designed the research, and supervised the report. Jun‐Yi Gao, Xiang Dai and Hao Wang contributed to the analysis. Chao‐Min Chen provided clinical advice.

## CONFLICT OF INTEREST STATEMENT

The authors declare no conflicts of interest.

## Data Availability

The data that support the findings of this study are available on request from the corresponding author. The data are not publicly available due to privacy or ethical restrictions.
